# Let-7 microRNA controls invasion-promoting lysosomal changes via the oncogenic transcription factor myeloid zinc finger-1

**DOI:** 10.1038/s41389-017-0014-6

**Published:** 2018-02-03

**Authors:** Siri Amanda Tvingsholm, Malene Bredahl Hansen, Knut Kristoffer Bundgaard Clemmensen, Ditte Marie Brix, Bo Rafn, Lisa B Frankel, Riku Louhimo, José Moreira, Sampsa Hautaniemi, Irina Gromova, Marja Jäättelä, Tuula Kallunki

**Affiliations:** 1 Cell Death and Metabolism, Center for Autophagy, Recycling and Disease, Danish Cancer Society Research Center, Strandboulevarden 49, 2100 Copenhagen, Denmark; 2 Biotech Research and Innovation Centre, University of Copenhagen, Copenhagen, Denmark; 3 Systems Biology Laboratory, Genome-Scale Research Program, Faculty of Medicine, University of Helsinki, Helsinki, Finland; 4 Department of Drug Design and Pharmacology, Faculty of Health and Medical Sciences, University of Copenhagen, Copenhagen, Denmark; 5 Breast Cancer Biology, Genome Integrity Group, Center for Autophagy, Recycling and Disease, Danish Cancer Society Research Center, Copenhagen, Denmark

## Abstract

Cancer cells utilize lysosomes for invasion and metastasis. Myeloid Zinc Finger1 (MZF1) is an ErbB2-responsive transcription factor that promotes invasion of breast cancer cells via upregulation of lysosomal cathepsins B and L. Here we identify let-7 microRNA, a well-known tumor suppressor in breast cancer, as a direct negative regulator of MZF1. Analysis of primary breast cancer tissues reveals a gradual upregulation of MZF1 from normal breast epithelium to invasive ductal carcinoma and a negative correlation between several let-7 family members and MZF1 mRNA, suggesting that the inverse regulatory relationship between let-7 and MZF1 may play a role in the development of invasive breast cancer. Furthermore, we show that MZF1 regulates lysosome trafficking in ErbB2-positive breast cancer cells. In line with this, MZF1 depletion or let-7 expression inhibits invasion-promoting anterograde trafficking of lysosomes and invasion of ErbB2-expressing MCF7 spheres. The results presented here link MZF1 and let-7 to lysosomal processes in ErbB2-positive breast cancer cells that in non-cancerous cells have primarily been connected to the transcription factor EB. Identifying MZF1 and let-7 as regulators of lysosome distribution in invasive breast cancer cells, uncouples cancer-associated, invasion-promoting lysosomal alterations from normal lysosomal functions and thus opens up new possibilities for the therapeutic targeting of cancer lysosomes.

## Introduction

Lysosomes are membrane-enclosed acidic organelles responsible for cellular clearance of damaged macromolecules and organelles^1^. In addition to these housekeeping functions, cancer cells can make effective use of lysosomes and their degradative enzymes to promote invasion and metastasis^2–4^. Malignant transformation and cancer progression to invasive disease are associated with altered lysosomal trafficking and increased expression and secretion of lysosomal cysteine cathepsins B and L^2,5–7^. When secreted to extracellular space, cathepsins modulate the microenvironment by cleaving and activating other invasion-promoting proteases, such as the urokinase plasminogen activator (uPA) system and matrix metalloproteases (MMPs), and by inactivating E-cadherin and CAM adhesion proteins expressed on the cell surface^5,8–10^. Accordingly, the lack of cathepsin B significantly delays and its overexpression further increases invasion and formation of lung metastases in the highly metastatic murine mammary tumor virus-polyoma middle T antigen (PyMT)-driven mammary cancer in mice^11, 12^. Similarly, the ErbB2-induced invasion of human breast cancer cell spheres in 3-dimensional (3D) Matrigel cultures depends on the increased expression and activity of cathepsin B^13^.

In addition to the increased lysosomal cathepsin activity, ErbB2-induced invasion of breast and ovarian cancer cells involves anterograde trafficking of lysosomes: in response to ErbB2 activation the lysosome distribution changes from a normal perinuclear or scattered distribution to the cell periphery^13,14^. Here they can secrete their contents, including cathepsin B, by lysosomal exocytosis and induce invasion-promoting intracellular and extracellular degradation^13,15,16^. ErbB2-induced cathepsin B expression is mediated by the transcription factor MZF1, which binds directly to the ErbB2-inducible enhancer element in the cathepsin B gene (*CTSB)*^13^. Malignant lysosomal distribution can be reverted by depletion of ErbB2 or cathepsins B and L. The possible regulatory role of MZF1 in the lysosome distribution has not been addressed before. In normal, non-cancerous cells the transcription factor EB (TFEB) is a master regulator of lysosomal biogenesis and exocytosis. It increases both cathepsin B expression and transport of lysosomes to the cell periphery^17,18^. TFEB is, however, not involved in the *CTSB* upregulation induced by ErbB2 in breast cancer cells^13^, suggesting that other transcription factors may regulate the anterograde trafficking of lysosomes in cancer cells.

MiRNAs of the let-7 family are among the miRNAs whose altered expression is most frequently associated with cancer^19^. Let-7 is upregulated during differentiation of normal cells and tissues^20^ and downregulated in poorly differentiated cancer tissues^21,22^. Its expression is strongly downregulated or even lost in many highly malignant cancers including advanced breast cancer^21^. In breast cancer-initiating cells, let-7 is one of the most consistently and significantly reduced miRNAs and it regulates all of their key tumorigenic features^21^, suggesting that let-7 may function as a tumor suppressor in breast cancer cells. Despite the correlation between the loss of let-7 and breast cancer aggressiveness, the mechanistic link between let-7 and breast cancer cell invasion and metastasis remains elusive.

Restoring the expression of let-7 family members has been suggested as a therapeutic tool against aggressive cancers^21,23^. In this study, we have used ectopic expression of let-7e, let-7g, and let-7d as a tool to study the effect of let-7 upregulation in invasive breast cancer cells. Here we describe a previously unnoticed link between let-7 and invasion by demonstrating that let-7e and let-7d can regulate cancer-induced invasion-promoting anterograde lysosome distribution in ErbB2-positive breast cancer cells by directly regulating the level of the oncogenic transcription factor MZF1.

## Results

### MZF1 expression is upregulated in human breast cancer

**Fig. 1:**
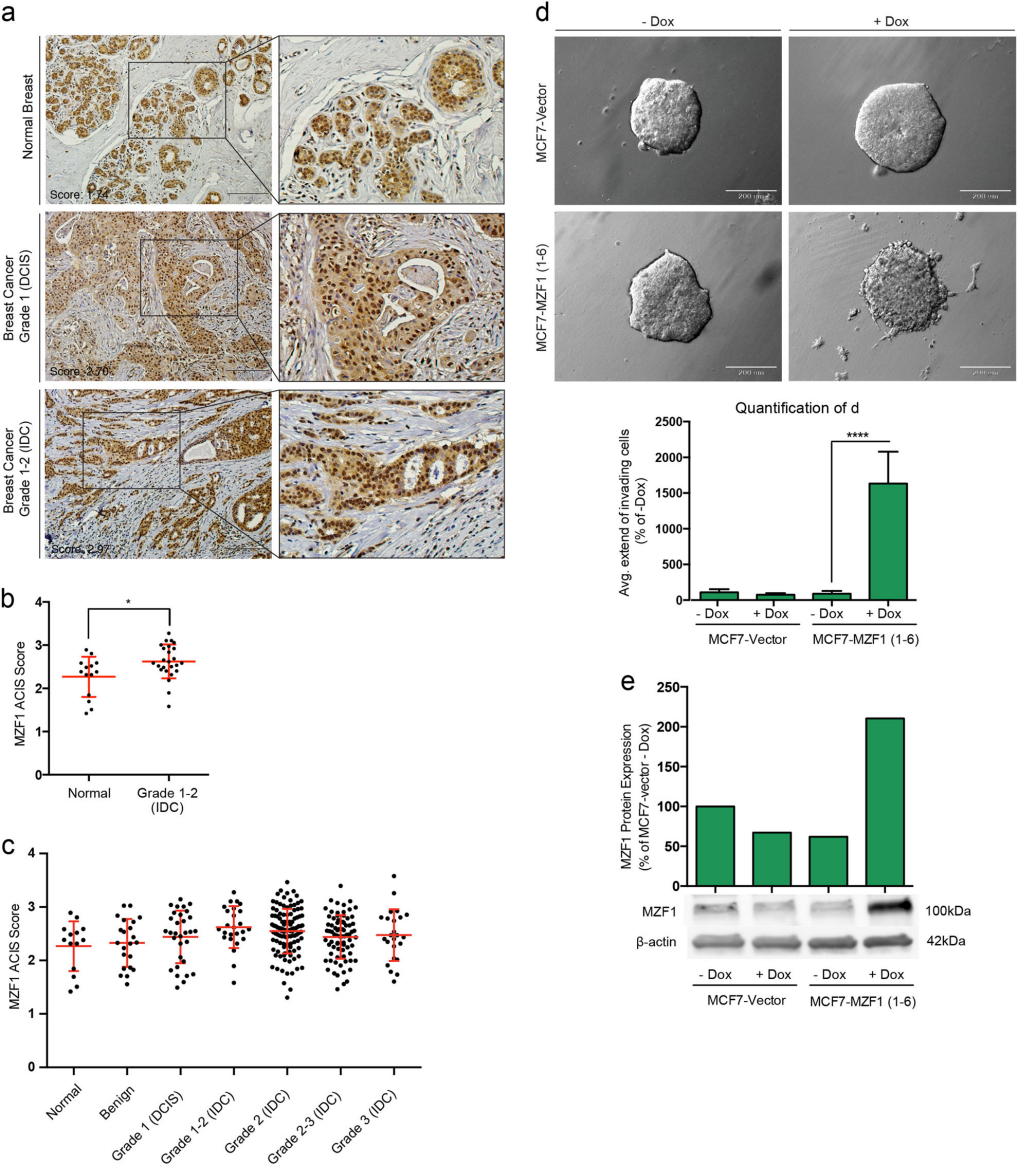
MZF1 expression is increased in primary breast cancer samples and its expression induces cancer cell invasion in poorly invasive breast cancer cells. **a** Examples of IHC staining of MZF1 (brown) in normal breast (upper panel), grade 1 breast cancer tissue (DCIS; middle panel), grade 1–2 breast cancer tissue (lower panel). Sections were counterstained with hematoxylin (blue) and images were taken with 20× magnification. **b** Association of MZF1 protein expression with tumor grade in primary breast cancer samples in TMAs including 321 primary samples of normal and breast cancer tissue. TMAs were analyzed by ACIS-assisted quantitative IHC and ACIS scores of duplicate cores were averaged and plotted against the breast cancer grade. The statistical difference in MZF1 expression between Normal and Grade 1–2 (IDC) is indicated by a * implicating a *p*-value < 0.05 according to a Mann–Whitney test. Mean values +/− SD is marked in red. **c** MZF1 expression in normal, benign and primary breast tumors of grade 1–3 from the same TMAs as in (**b**). **d** Invasion of MCF7 cells stably transfected with a doxycycline-inducible expression vector containing MZF1 (MCF7-MZF1 1–6) or an empty vector (MCF7-vector) in 3D Matrigel in the absence (−Dox) or presence of doxycyline (+Dox). Images were taken with 10x magnification. Quantification of the average extend of outgrowth in (**d**) was done using ImageJ. For each of 4 spheres per treatment, the 10 greatest distances traveled by invading cells were estimated and a mean outgrowth was calculated for each treatment. Data presented is an average of 2 independent experiments and as % of non-induced control (−Dox) and the comparison of −Dox and +Dox in MCF7-MZF1 1–6 cells is assigned with **** indicating *p* < 0.0001 in a Welch’s *t*-test. **e** Immunoblot analysis of MZF1 expression in MCF7-vector and MCF7-MZF1 1-6 (+Dox) and without (−Dox) doxycycline. β-actin was used as a control of equal loading. The immunoblot from a single control experiment was quantified using ImageJ, normalized to β-actin and presented as % of MCF7-vector −Dox

We compared MZF1 protein expression in tissue microarrays (TMAs) containing 321 samples of normal breast tissue and different grades of primary breast cancer by quantitative immunohistochemistry (IHC). MZF1 was expressed predominantly in the nucleus of both normal ductal epithelial cells and cancer cells (Fig. 1a). MZF1 expression was increased when comparing normal tissues to invasive ductal carcinoma (IDC; grades 1–2) (Fig. 1b; Supplementary Figure S1a). In samples of more advanced IDC (grades 2–3), the mean MZF1 expression remained increased when compared with normal breast epithelium samples (Fig. 1c). The specificity of the MZF1 antibody was verified by IHC staining of paraffin sections of MCF7 breast cancer cells expressing doxycycline-inducible MZF1 (Supplementary Figure S1b) and with a peptide competition assay (Supplementary Figure S1c). MZF1 expression was increased in a panel of breast cancer cells lines including MCF7, BT474, SK-BR-3, MDA-MB-231, MDA-MB-436, and MDA-MB-468 when comparing with non-cancerous, immortalized MCF10A cells (Supplementary Figure S1d). Taken together these results show that MZF1 is expressed in differentiated mammary epithelia and that its expression is increased in primary human breast cancer.

### MZF1 renders breast cancer cells invasive in vitro

To investigate whether MZF1 could have a direct role in the breast cancer invasiveness, we stably expressed a doxycycline-inducible MZF1 expression construct in poorly invasive MCF7 breast cancer cells that exhibit low expression of all endogenous, ligand-induced ErbB receptors^24^. We studied the invasiveness of doxycycline-inducible MCF7-MZF1 1–6 clone in a 3D Matrigel invasion assay (Fig. 1d; Supplementary Figure S1e). The doxycycline-induced 3-fold increase in MZF1 protein expression (Fig. 1e) was associated with increased invasiveness of MCF7-MZF1 1–6 cells (Fig. 1d). 1.5-fold increase in the expression of MZF1 obtained with another cell line expressing doxycycline-inducible MZF1 (MCF7-MZF1 1–9) treated with 5 nM epidermal growth factor (EGF) to induce ErbB signaling, resulted in modest increase in the invasion of the MCF7 spheres (Supplementary Figure S1e). Notably, doxycycline and EGF had no effect on the invasiveness of the MCF7-vector cells (Fig. 1d; Supplementary Figure S1e). Thus, the increased expression of MZF1 can promote invasion of poorly invasive MCF7 cells. Conversely, we have previously shown that invasion of breast cancer cells expressing high levels of ErbB2 could be inhibited by reducing MZF1 expression with MZF1 siRNA^13^.

### Let-7 miRNAs regulate MZF1 expression directly via the 3’-UTR of MZF1

**Fig. 2:**
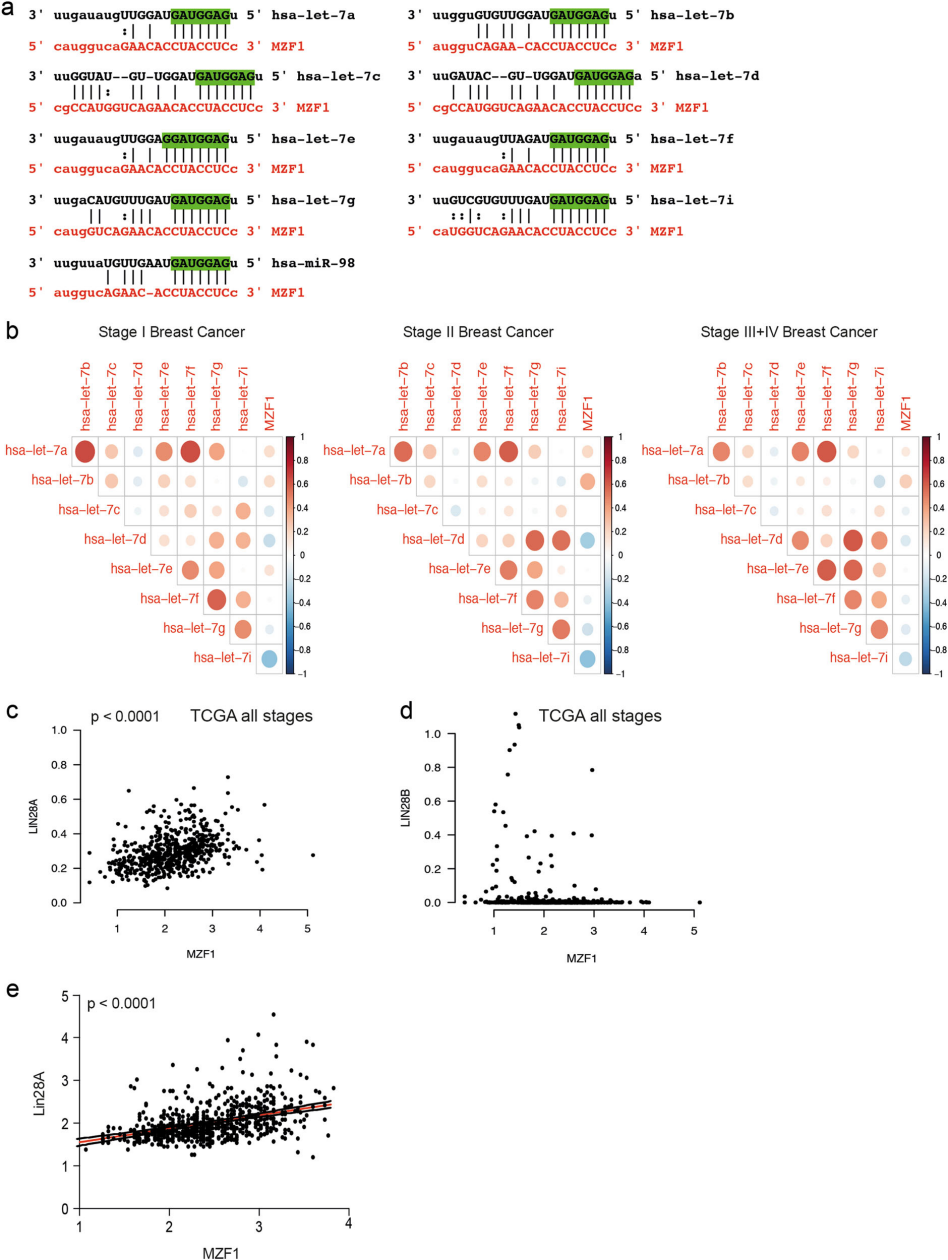
The MZF1 3′-UTR contains a putative let-7 target site and MZF1 expression correlates negatively with several let-7 family members and positively with LIN28A in primary breast cancer samples. **a** Alignment of 9 let-7 family members to their putative target site in the MZF1 3′-UTR (red). All let-7 family members are predicted to bind to the target site via a 7mer seed region (green), with the exception of let-7e binding with an 8mer seed region. **b** Correlation plots for the correlation between let-7 and MZF1 RNA expression in primary breast cancer samples included in TCGA data. Pairs that show positive correlation are depicted with red circles. Pairs that show negative correlation are depicted with blue circles. The Spearman correlation coefficient is depicted using the radius of the circle: the larger the coefficient, the larger the radius. **c** Correlation plots of MZF1 and LIN28A expression in TCGA data (all stages). Highest 6% expressing LIN28A samples have been omitted from the figure to better show the correlation. The correlation is assigned with *** indicating *p* < 0.001. **d** Correlation plots of MZF1 and LIN28B expression in TCGA (all stages). **e** Association between MZF1 and Lin28A protein expression quantified from the same TMAs as described in Fig. 1a–c. The Spearman correlation is significant with *p* < 0.0001

In search for mechanisms responsible for the upregulation of MZF1 during breast cancer progression to IDC, we screened the MZF1 3′-untranslated region (UTR) for putative miRNA binding sites in microRNA.org (http://www.microrna.org/microrna/home.do); a miRNA target prediction resource based on the MiRanda algorithm^25^. microRNA.org predicted a single putative binding site for the let-7 family of miRNAs in the 3′-UTR of MZF1 with probability of downregulation (mirSVR score: −0.1253 to −0.1229) and miRNA conservation (PhastCons score: 0.3954) (Fig. 2a). Notably, no other miRNA target sites were found in the 3′-UTR of MZF1 with these criteria. The let-7 family members share a 7-nucleotide seed region (5′-GAUGGAG-3′) with complete complementarity to the MZF1 target sequence. In let-7e the seed region is extended to 8-nucleotides (Fig. 2a). Using mRNA expression array data (*n* = 547) and miRNA sequencing data (*n* = 697) from The Cancer Genome Atlas (TCGA; https://cancergenome.nih.gov/) database, we correlated MZF1 mRNA expression with let-7 miRNA expression employing a Spearman’s rank correlation analysis. Supporting the miRNA.org prediction, we found that the expression of 5, 6, and 7 out of 11 studied let-7 miRNA family members correlated negatively with the expression of MZF1 in grade I, II and III + IV, respectively (Fig. 2b). One mechanisms proposed to be responsible for the downregulation of let-7 in breast cancer is the upregulation of the well-studied inhibitors of let-7 processing; lin28A and lin28B^26–28^. Thus, we utilized the mRNA expression array data from TCGA to investigate the correlation between MZF1 and lin28A and lin28B mRNA (LIN28A and LIN28B) in breast cancer and found that MZF1 expression correlates positively with the expression of lin28A, but not lin28B (Fig. 2c, d). This suggests that lin28A could be involved in the regulation of MZF1 expression via let-7 in breast cancer. Supporting this, quantitative IHC of the breast cancer TMAs used in Fig. 1a–c revealed a significant positive correlation between lin28A and MZF1 protein expression (Fig. 2e).

**Fig. 3:**
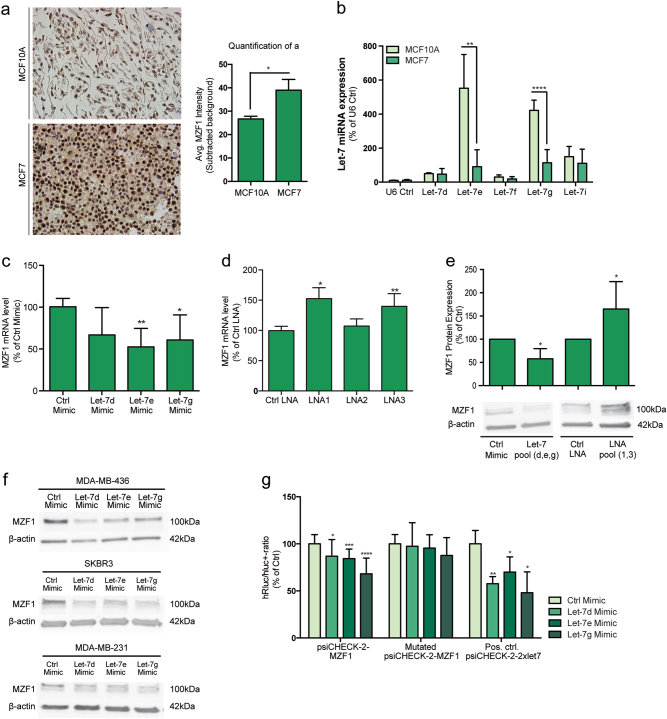
Let-7 miRNAs regulate MZF1 expression by directly binding to its 3′-UTR. **a** Representative IHC staining of MZF1 in sections made from formalin-fixed, paraffin-embedded (FFPE) MCF10A and MCF7 cell pellets. Sections were counterstained with hematoxylin (blue) and images were taken with 40x magnification. MZF1 expression in MCF10A and MCF7 cells was quantified using ACIS-assisted analysis. **b** Let-7 expression in MCF10A and MCF7 cells. Expression of let-7d, let-7e, let-7f, let-7g, and let-7i was quantified using TaqMan miRNA assay and qPCR. The relative miRNA quantity was calculated by 2-ΔCt using U6 RNA as an internal reference. U6 RNA was expressed at similar levels in MCF10A and MCF7 cells. Data are presented as the means of two independent experiments with three technical replicates ± SD. The comparison of MCF10A and MCF7 cells according to let-7e- and let-7g-expression is assigned with ** indicating *p* < 0.01 and **** indicating *p* < 0.0001 in Welch’s *t*-test. **c** The effect of let-7d, let-7e and let-7 g mimics (20 nM) on MZF1 mRNA level relative to non-targeting control mimic in MCF7 cells. MZF1 mRNA expression was quantified by RT-qPCR with PPIB as reference gene. Data are presented as the mean of six (let-7d and let-7e) and five (let-7g) independent experiments ± SD and significance is indicated with * for *p* < 0.05 in a Welch’s *t*-test. **d** The effect of miRNA inhibitors LNA1, LNA2 and LNA3 (20 nM) targeting different let-7 family members on MZF1 mRNA expression relative to an LNA negative control in MCF7 cells. Data are presented as the mean of three independent experiments ± SD and significance is indicated with * and ** for *p* < 0.05 and *p* < 0.01 in a Welch’s *t*-test, respectively. **e** Immunoblot-analysis of MZF1 protein expression in response to a let-7 pool (let-7d, let-7e, and let-7g, 20 nM) or LNA pool (LNA1 and LNA3, 20 nM). β-actin was used to control the equal loading. The immunoblot is a representative of three independent blots that were quantified using ImageJ. Data are presented as the mean of three experiments ± SD relative to control. Significance is indicated with * for *p* < 0.05 in a Welch’s t-test. **f** Immunoblot-analysis of MZF1 protein expression in response to a transfection with 20 nM of let-7d, let-7e, and let-7 g mimic and a non-targeting control in SKBR3, MDA-MB-231 and MDA-MB-436 cells. β-actin was used to control the equal loading. The immunoblot is a representative of three independent blots. **g** Luciferase reporter assay measuring the hluc + − and hRluc-response of psiCHECK-2-MZF1, Mutated psiCHECK-2-MZF1 and a positive control psiCHECK-2–2xlet-7 to mimics of let-7d, let-7e and let-7g (20 nM). Data are presented as the mean of 12 independent experiments for psiCHECK-2-MZF1 and mutated psiCHECK-2-MZF1 and 4 independent experiments for psiCHECK-2–2xlet7. Data are presented as the mean of experiments ± SD relative to control mimic and significance is indicated with *, **, ***, and **** for *p* < 0.05, 0.01, 0.001, and 0.0001 in a Welch’s *t*-test, respectively

To study the effects of let-7 overexpression and inhibition of MZF1 expression in breast cancer cells, we used MCF7 cells that express both MZF1 (Supplementary Figure S1d)^13^ and most of the let-7 family members^29^. Consistent with the expression pattern of MZF1 in the TMA described above (Fig. 1b) and in breast cancer cell lines (supplementary Figure S1d), we found MZF1 expression to be higher in MCF7 breast cancer cells than in the non-cancerous, immortalized MCF-10A breast epithelial cells (Fig. 3a). By quantifying the expression of five representatives of let-7 family members let-7d, let-7e, let-7f, let-7g, and let-7i in these cell lines, we found that the expression of let-7e and let-7g was significantly lower in MCF7 than in MCF10A cells (Fig. 3b).

We chose to continue cellular studies with three representatives of let-7 family: let-7d, let-7e, and let-7g. This choice was based on the results above and on earlier publications that found let-7d and let-7g to be involved in breast cancer cell invasion^30,31^. Additionally, let-7e was chosen due its extended MZF1-complimentary seed region (Fig. 2a). Extended seed regions have been shown to enhance the efficiency of target down-regulation^32^. Since the putative let-7 target site in the MZF1 3′UTR has complete complementarity in the seed region and additional partial complementarity in the 3′-end to all members of let-7 family (Fig. 2a), we assumed that the site is likely regulated by all or none of the members of the let-7 family^33^.

In order to investigate whether let-7 overexpression could reduce MZF1 expression in MCF7 cells, we transfected MCF7 cells with 20 nM of let-7d, let-7e, and let-7g miRNA mimics. Ectopic expression of let-7e and let-7g significantly decreased MZF1 mRNA expression when comparing with a non-targeting control mimic (Fig. 3c). The effect of let-7d on MZF1 mRNA expression was not significant (Fig. 3c). To investigate the effect of let-7 inhibition on MZF1 expression, we used locked nucleic acid (LNA) miRNA inhibitors. We transfected MCF7 cells with 20 nM of LNA1 targeting let-7a, let-7b, let-7c, let-7d, and let-7e, LNA2 targeting only mir-98 and LNA3 targeting let-7f, let-7g and let-7i. LNA1 and LNA3, but not LNA2, increased MZF1 mRNA expression significantly when comparing with a non-targeting control LNA (Fig. 3d). Supportively, transfection of MCF7 cells with a pool of the let-7d, let-7e, and let7-e mimics and a pool of LNA1 and LNA3 decreased and increased the MZF1 protein expression, respectively (Fig. 3e). Additionally, transfection of let-7 mimics d, e and g into breast cancer cell lines SK-BR-3, MDA-MB-436, and MDA-MB-231 resulted in a reduction in MZF1 protein expression in most of the samples (Fig. 3f), suggesting a more general role of the regulatory relationship between these let-7 family members and MZF1 in breast cancer cells.

To investigate whether the effect of let-7 on MZF1 expression is a result of direct interaction between let-7 miRNAs and the MZF1 3′-UTR, we subcloned the MZF1 3′-UTR containing the let-7 target site (psiCHECK-2-MZF1) and its mutated version (Mutated psiCHECK-2-MZF1; CTACCTC to CAAGCAC) into a psiCHECK-2 luciferase expression vector. Luciferase assays showed that expression of mimics of let-7d, let-7e, and let-7g significantly decreased the luciferase activity of the psiCHECK-2-MZF1, but not the mutated psiCHECK-2-MZF1 in MCF7 cells (Fig. 3g). As a positive control, we used a psiCHECK-2 expression vector containing two synthetic let-7 binding sites (Pos. ctrl. psiCHECK-2–2xlet7) (Fig. 3g).

Taken together these results show that let-7d, let-7e, and let-7g can regulate MZF1 expression in various breast cancer cells directly via a functional let-7 target site in the MZF1 3′-UTR.

### Let-7 regulates invasion and lysosomal distribution of ErbB2-expressing breast cancer cells via MZF1

**Fig. 4:**
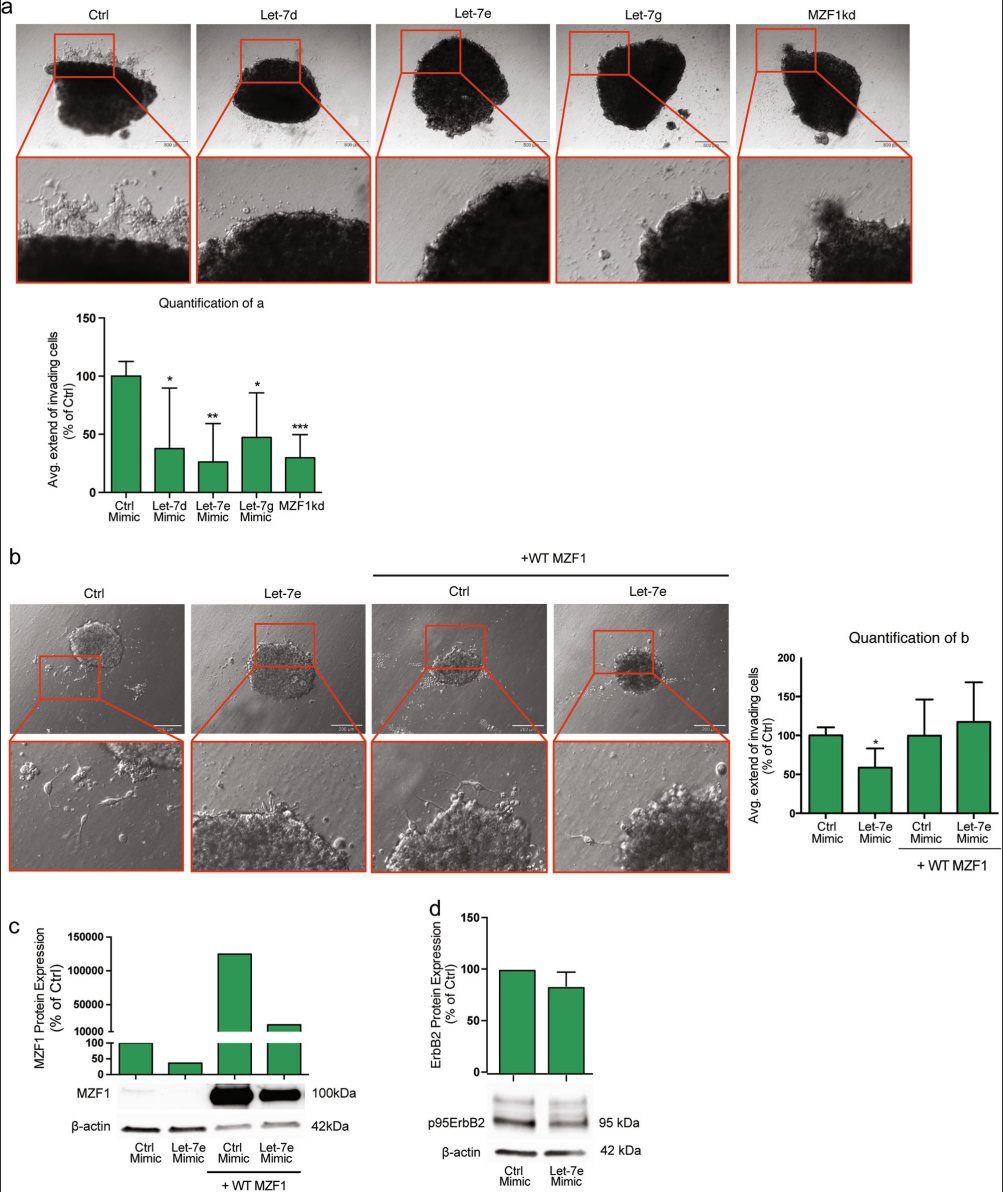
Let-7 miRNAs inhibit invasion of invasive breast cancer cells by reducing MZF1 expression. **a** Invasion of MCF7-p95ΔNErbB2 cells in 3D Matrigel upon transfection with 20 nM control, let-7d, let-7e, and let-7g mimic and MZF1 siRNA. Images of the invasive spheres were taken at 10× magnification. Quantification of the average extend of outgrowth in (**a**) was done using ImageJ. For each of 5 spheres per treatment, the 10 greatest distances traveled by invading cells were estimated and a mean outgrowth was calculated for each treatment. Data are presented as % of control mimic and the effect of let-7e and MZF1kd is assigned with *, **, and *** indicating *p* < 0.05, *p* < 0.01, and *p* < 0.001, respectively, in a Welch’s *t*-test. **b** Invasion of MCF7-p95ΔNErbB2 cells in 3D Matrigel upon transfection with a control and 20 nM let-7e mimic in the absence and presence of transiently expressed WT MZF1 lacking the 3′-UTR. Images of the invasive spheres were taken with 10× magnification. Quantification is done as described in **a**. Data are presented as % of control and the effect of let-7e is assigned with * indicating *p* < 0.05 in a Welch’s *t*-test. **c** Immunoblot analysis of MZF1 protein expression in MCF7-p95ΔNErbB2 cells transfected with 20 nM of control and let-7e mimic in the absence and presence of transient overexpression of WT MZF1. β-actin was used to control for equal loading. The immunoblot was quantified using Image Studio Lite. Data are presented as relative to control mimic treatment in the absence of transient WT MZF1 overexpression. **d** Immunoblot analysis of ErbB2 protein expression in MCF7-p95ΔNErbB2 cells response to forced let-7e expression (20 nM). β-actin was used to control the equal loading. The immunoblot is a representative of three independent experiments that were quantified using Image Studio Lite. Data are presented as the mean of experiments ± SD relative to control. The effect was non-significant according to a Welch’s *t*-test

In order to study the effect of let-7 miRNAs on breast cancer cell invasion, we used MCF7 cells rendered highly invasive by inducible expression of constitutively active 95kDa N-terminally truncated form of ErbB2 (MCF7-p95ΔNErbB2)^13,34^. Forced expression of let-7d, let-7e, and let-7g decreased the invasive growth of MCF7-p95ΔNErbB2 cells in 3D Matrigel (Fig. 4a). Let-7e was the most efficient of the tested let-7 family members, inhibiting invasion to the same extend as MZF1-targeting siRNA (MZF1kd), when comparing with a non-targeting control (Fig. 4a). The ectopic expression of let-7 mimics had no significant effect on survival of the MCF7-p95ΔNErbB2 cells within the timeframe relevant for this assay (Supplementary Figure S2a).

In order to investigate whether the effect of let-7 on invasion is mediated by MZF1, we performed a rescue experiment using co-expression of let-7e mimic and a plasmid expressing wild type (WT) MZF1 lacking the 3′-UTR. We chose to rescue the effect of let-7e since this was the let-7 mimic with the most prominent effect on invasion in these cells. Indeed, transient overexpression of this WT MZF1 could rescue the inhibitory effect of let-7e on invasion, suggesting that the effect is mediated by MZF1 (Fig. 4b, c). Transfection of MCF7-p95ΔNErbB2 cells with the let-7e mimic did not significantly affect the expression of p95ErbB2 (Fig. 4d).

**Fig. 5:**
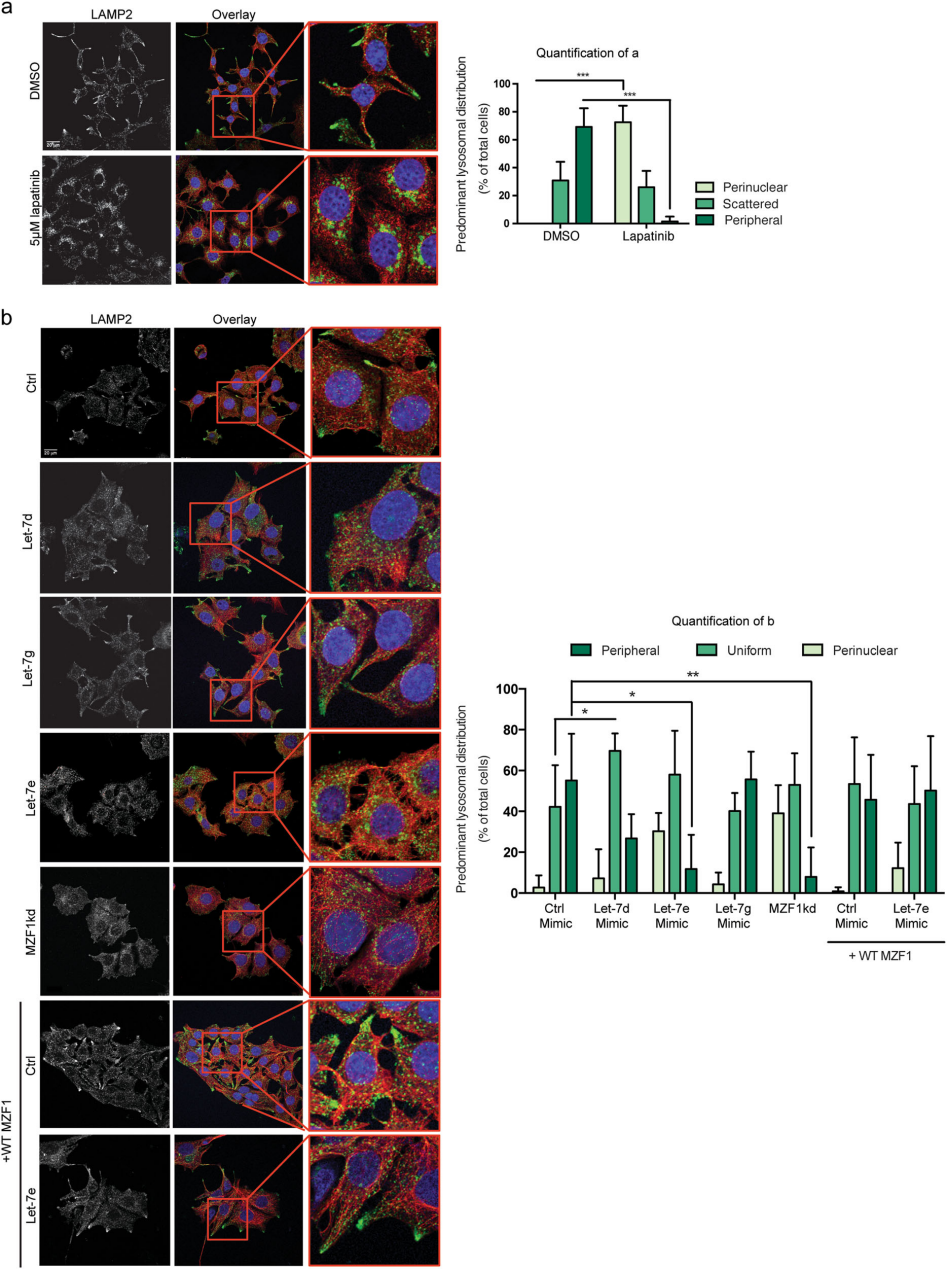
Let-7 miRNAs reverse ErbB2-induced peripheral distribution of lysosomes by reducing MZF1 expression. **a** Confocal immunofluorescence microscopy images of the lysosomal distribution in MCF7-p95ΔNErbB2 cells treated with DMSO or 5 μM lapatinib for 24 h. After treatment, cells were fixed and stained for the lysosomal membrane protein LAMP2 (green), cytoskeletal α-tubulin (red) and nucleus with Hoechst (blue). Quantification of (**a**) was done by characterizing the lysosomal distribution as predominantly perinuclear, scattered or peripheral in 8–15 cells per 63× image in five images per treatment. Data represents the mean percentage distribution of five images ± SD. The effect of lapatinib on the peripheral pool of lysosomes is assigned with *** indicating *p* < 0.001 in a Welch’s *t*-test. **b** Confocal immunofluorescence microscopy images of the lysosomal distribution in MCF7-p95ΔNErbB2 cells transfected with 20 nM of a non-targeting control, let-7d, let-7e, and let-7g mimic and MZF1 siRNA (MZF1kd) and the non-targeting control and let-7e mimic in the presence of transiently expressed WT MZF1 lacking the 3′-UTR. Quantification of (**b**) was done as described in for (**a**). The effect of let-7 mimics and MZF1 siRNA on the peripheral pool of lysosomes is assigned with *, **, and *** indicating *p* < 0.05, *p* < 0.01, and *p* < 0.001 in a Welch’s *t*-test. The data presented in A and B are representative of three independent experiments

In MCF7-p95ΔNErbB2 cells, ErbB2 signaling induces a malignant cellular morphology characterized by the appearance of long cellular protrusions and a dramatic redistribution of lysosomes to the cell periphery^13,34^. We have previously shown that lapatinib, an EGFR/ErbB2 kinase inhibitor, inhibits invasion and alters the lysosomal distribution from predominantly peripheral to predominantly perinuclear in ErbB2-positive ovarian cancer cells^14^. Confocal immunofluorescent microscopy of the lysosomal distribution (LAMP2 staining) in MCF7-p95ΔNErbB2 cells revealed a similar lapatinib-induced retrograde trafficking of lysosomes (Fig. 5a). The lapatinib concentration used in this assay did not affect the survival of the cells (Supplementary Figure S2b). Supporting the role for MZF1 in ErbB2-induced anterograde trafficking of lysosomes, its depletion by let-7e and let-7d mimics or *MZF1* siRNA reduced the proportion of peripheral lysosomes while increasing that of perinuclear lysosomes (Fig. 5b). Forcing the expression of let-7g had no effect on the predominant lysosomal localization (Fig. 5b). Since the effect on lysosome distribution in these cells was most prominent when overexpressing the let-7e mimic, we performed a rescue experiment using let-7e and ectopically expressed MZF1. Similar to the effect on invasion, the effect of let-7e on the lysosomal distribution could be rescued by the transient overexpression of *MZF1* lacking the 3′-UTR (Fig. 5b).

These results indicate that ectopic expression of let-7 family members can effectively counteract MZF1-mediated invasion and related peripheral distribution of lysosomes.

## Discussion

MZF1 is a zinc-finger transcription factor expressed in myeloid progenitor cells where its function is to regulate hematopoietic differentiation^35^. MZF1 is also expressed in various epithelial cancers, including breast cancer, where it promotes tumor progression^13,36–45^. The knowledge of its role in breast cancer has been limited to the fact that it is ErbB2-responsive and that it contributes to invasion by upregulating *CTSB* and *PRKCA* (protein kinase C alpha) expression in various breast cancer cell lines^13,39,45^. In this study, we show that MZF1 expression is increased during breast cancer progression from normal epithelium to DCIS and IDC and that increased MZF1 expression can induce invasion of poorly invasive, MCF7 breast cancer cells in 3D Matrigel cultures. Thus, MZF1 may contribute to the progression of mammary ductal epithelial cells to abnormal cells confined in the duct, and finally to invasive breast cancer. By identifying MZF1 as a novel target of let-7, this study further suggests that the lack of let-7 suppression on MZF1, could result in MZF1 upregulation and thus contribute to oncogenesis and breast cancer progression.

Despite extensive evidence that loss of let-7 expression associates with invasive and metastatic disease and poor patient outcome in breast cancer^30,31,46,47^, the factor/s linking these two are still not understood. In this study, we present a functional link between let-7 family members and breast cancer cell invasion, by identifying MZF1 as a novel target of let-7 and by connecting let-7 to the invasion-promoting lysosomal distribution of ErbB2-positive breast cancer cells via MZF1. Previous studies have identified additional oncogenic let-7 targets. Among these are the High Mobility Group 1 and 2 A (HMGA1 and 2 A) protein, BACH1, estrogen receptor α (ERα), the proto-oncogenes c-Myc and Ras^46,48–52^. The contribution of these let-7 targets to the invasion of ErbB2-positive breast cancer cells has not been addressed. However, the fact that the ectopic expression of MZF1 lacking the 3′UTR can fully rescue the let-7-mediated inhibition of invasion of ErbB2-positive cells in this study, suggests that the invasion-inhibiting function of let-7 in these cells could be mediated by MZF1. Moreover, our finding that in breast cancer cells the level of MZF1 and consequently, the MZF1-controlled anterograde lysosome trafficking can be regulated by let-7, identifies a previously unnoticed function for this well-studied miRNA family.

Here we conclude that MZF1 expression is needed for the ErbB2-induced, invasion-promoting anterograde lysosomal trafficking in breast cancer cells. This adds to its previously reported role as a transcriptional regulator of cathepsins B and L upon ErbB2 activation^13^. Thus, MZF1 performs similar functions in breast cancer cells as TFEB, also known as a master regulator of the Coordinated Lysosomal Expression and Regulation (CLEAR) network, has in normal cells. TFEB function is negatively regulated by mTOR and ERK. Phosphorylation of TFEB by mTOR and ERK kinases masks the nuclear localization signal of TFEB and sequesters it in the cytosol, where it cannot activate the expression of its target genes^17,53,54^. Thus, TFEB is most likely not involved in the regulation of the ErbB2-induced, invasion-promoting lysosomal alterations in breast cancer cells, since ErbB2 activation leads to the activation of the mTOR and MAPK-ERK signaling pathways^55^. Supportively, TFEB was found not to be involved in the ErbB2-induced expression of CTSB, a central lysosomal hydrolase belonging to the CLEAR network^13^. This data altogether suggests that MZF1 can, at least partially, take over some of the functions of TFEB in breast cancer cells.

One potentially important implication of this study is the identification of MZF1 as the factor that can uncouple the oncogene-induced lysosomal alterations from the TFEB-regulated CLEAR network, which is highly important for the normal cellular wellbeing. The fact that these two processes can be regulated by different factors in cancer cells versus normal cells makes MZF1 and the lysosomal alterations it induces attractive targets for anti-cancer drug development. Thus, discovering and understanding how the lysosomal compartment in cancer cells differs from that of normal cells and what regulates these differences, will allow the intervention of invasion-promoting processes of cancer cells without interfering with the cellular clearance and wellbeing of normal cells.

## Materials and methods

### Tissue Culture and cell lines

Human breast cancer cell lines MCF7^56^ were grown in RPMI1640 Glutamax^TM^; SK-BR-3, MDA-MB-231, MDA-MB-436 and MDA-MB-468 (ATCC, Manassas, VA, USA) in DMEM GlutaMAX^TM^; BT474 (ATCC) in DMEM/F-12 (GIBCO^TM^, Invitrogen, Carlsbad, CA, USA) supplemented with 6 or 10% of heat inactivated fetal bovine serum (FBS) (GIBCO^TM^, Invitrogen). The human breast epithelial cell line MCF10A (ATCC) was cultured in DMEM/F-12 (GIBCO^TM^, Invitrogen) supplemented with 5% horse serum (GIBCO^TM^, Invitrogen), 10 µg EGF (Sigma-Aldrich E-H127, St. Louis, MO, USA), 250 µg hydrocortisone (VWR CALB3867-1, Radnor, PA, USA), 50 µg CholeraToxin (Sigma-Aldrich C-8052), and 5 mg insulin (Sigma-Aldrich I-9278). All the cells were grown in the presence of 100,000 units/mL penicillin and 12.5 µg/mL streptomycin (Invitrogen). Doxocycline inducible MZF1 expressing cells were prepared by amplifying the coding sequence of full-length MZF1 by PCR using a QIAGEN LongRange PCR kit (QIAGEN, Hilden, Germany) and cloning the DNA fragment into the EcoRI site of the pVLX-Tight-Puro-Tet-On Advanced vector. Construct was confirmed by sequencing. The inducible MZF1 expression vector was stably transfected into MCF7 and the culture media was supplemented with 200 µg/mL G418 and 1 µg/mL puromycin to obtain single cell MZF1 expressing clones 1–6 and 1–9. MCF7 p95∆N-ErbB2 cells were cultured and the expression of ErbB2 was induced as described previously^13,34^. All cell lines were kept at 37 °C in a humidified atmosphere of 5% CO_2_.

### Immunohistochemical (IHC) staining of formalin-fixed paraffin-embedded (FFPE) cell lines and tissues

Tissue microarrays (TMAs) were purchased from Pantomics (BRC961, BRC962 BRC1501, BRC1502, BRC1503), Richmond, CA, USA). Additional tissue samples of normal breast tissue were produced and processed as previously published^57^. FFPE cell-blocks were prepared as in Cabezon et al.^58^. Five micrometer sections were stained with primary antibody against MZF1 (Abcam ab64866, 1:1000, Cambridge, UK) or Lin28A (Abcam ab46020, 1:1000). The sections were scanned and assessed in an ACIS^®^ III Instrument (DAKO). IHC quantification was done by ACIS III-assisted analysis. The stained sections were scanned and brown intensity was quantified with manually placed 40× regions (3 per section/core) and a color threshold customized for MZF1.

### Transfections

To force and inhibit the expression of let-7 miRNAs we used hsa-let-7d, has-let-7e and Negative Control 2 (Mission® microRNA, Sigma-Aldrich) and locked nucleic acids (LNAs) hsa-let7 F1 (LNA1) targeting hsa-let-7a, hsa-let-7b, hsa-let-7c, hsa-let-7d and hsa-let-7e, hsa-let-7 F2 (LNA2) targeting hsa-mir98, hsa-let-7 F3 (LNA3) targeting hsa-let-7f, hsa-let-7g and hsa-let-7i and, negative control B (miRCURY^®^ LNAs, Exiqon, Vedbaek, Denmark), respectively, in a final concentration of 20 nM. As a control, siRNA targeting MZF1 (# sc-45714A; Santa Cruz Biotechnology, Santa Cruz, CA, USA) was also included.

Reverse transfection with mimics, LNAs and siRNA (20 nM) were performed in 6-well or 96-well culturing plates with RNAiMAX (Invitrogen^TM^) according to manufacturer’s protocol. Cells were harvested after 24, 48, and 72 h for invasion, RNA and protein, respectively. Transfections with a pcDNA3.1 vector containing the WT MZF1 were performed in 6-well culturing plates using Fugene HD (Promega, Madison, WI, USA) as a transfection reagent according to manufacturer’s protocol.

### RNA isolation and quantification

Total RNA was isolated and purified using TRIzol Reagent (Invitrogen) followed by phenol-chloroform extraction (Sigma-Aldrich) and precipitation. Let-7 miRNAs were quantified from RNA samples using a TaqMan® MicroRNA Assay (Life Technologies Carlsbad, CA, USA) according to manufacturer’s protocol. For quantitative PCR to determine the mRNA levels of MZF1, RNA was isolated using Nucleospin^TM^ Total RNA Isolation Kit (Macherey-Nagel, Düren, Germany) according to the manufacturers protocol. The cDNA was synthesized from 1 µg RNA with Oligo(dT) primers using a TaqMan^TM^ Reverse Transcription Kit (Roche Diagnostics, Basel, Switzerland). QPCR analysis was performed using SYBR Green QPCR reagents (Agilent Technologies, Santa Clara, CA, USA) in a 7500 Fast Real-Time PCR System (Applied Biosystems, Foster City, CA, USA). The expression of target genes was normalized to the expression of the housekeeping gene Cyclophilin B (PPIB). The Pfaffl method, which takes primer efficiencies into account, was used to calculate Relative mRNA levels were calculated using the Pfaffl method^59^. Following primers were used for quantitative PCR:

MZF1 forward: 5′- TGG GCC TCT AGC TGC CAC CC-3′ (efficiency 1.866076)

MZF1 reverse: 5′- GGT CCC ACA TCT CTG GGC CTC C-3′ (efficiency 1.866076)

PPIB forward: 5′- GGG AGA TGG CAG AGG AGG AAA −3′ (efficiency 1.739843)

PPIB reverse: 5′-TGG GAG CCG TTG GTG TCT TTG-3′ (efficiency 1.739843)

### Immunoblotting

Immunoblotting was performed as described before^13,14^. The primary antibodies used were anti-MZF1 (Abcam, ab64866), anti-ErbB2 (Thermo Scientific, ab-17, Waltham, MA, USA) and anti-β-actin (Sigma-Aldrich, A2228). Images were quantified using Image J or Image Studio Lite.

### Luciferase assay with psiCHECK^TM^-2 reporter

The MZF1 3′UTR region from a pMirTarget Reporter (OriGene^®^ Technologies, Rockville, MD, USA) was transferred to a psiCHECK-2 vector (Promega) by digestion with NotI and SgfI followed by ligation using the Rapid DNA Ligation kit (Roche Diagnostics) according to the manufacturers protocol. Correct insertion of the MZF1 3′UTR into the psiCHECK-2 vector was confirmed by sequencing. Let-7 mimics were reverse transfected into MCF7 cells as previously described, following forward transfection with psiCHECK-2-MZF1 after 24 h using Fugene HD (Promega) according to manufacturer’s protocol. Luciferase assays were performed using Dual-luciferase^TM^ Reporter Assay System (Promega) and measured with a Varioskan^®^ Flash Spectral Scanning Reader (Thermo Scientific). Renilla luciferase activities were normalized to firefly luciferase activities.

To mutate the let-7 target site in the psiCHECK-2-MZF1, three substitutions were introduced in the seed region (CTACCTC- > C**A**A**G**C**A**C) by site-directed mutagenesis. Mutagenesis primers were designed using the QuikChange^®^ Primer Design program from Agilent Technologies (https://www.genomics.agilent.com/primerDesignProgram.jsp) (Mut forward: 5′- GCC ATG GTC AGA ACA CCA AGC ACC CCT GGT TAT TGT GAG-3′ and Mut reverse: 5′- CTC ACA ATA ACC AGG GGT GCT TGG TGT TCT GAC CAT GGC-3′). The mutations were introduced using PfuUltra II Fusion HS DNA polymerase (Agilent Technologies) and the Dpn I restriction enzyme (10 U/µL) at 37 °C for 1–2 h, to remove the parental supercoiled dsDNA vector. The resulting mutated psiCHECK2-MZF1 was verified by sequencing. A psiCHECK-2 vector containing a tandem let-7 target site (psiCHECK-2–2xlet-7) was included as a positive control (Addgene plasmid #20929, Cambridge, MA, USA).

### 3D Invasion assay

A day after transfection, 3D cell spheres were made with a hanging drop method^13^ or with a spinning method^60^ in RPMI supplemented with 6% heat inactivated FCS. 20–24 h later spheres were embedding into Matrigel (Growth factor reduced, BD Biosciences no. 354263, San Jose, CA, USA) containing 1.5% FCS and incubated in surrounding RPMI supplemented with 10% of fresh FCS for maximal utilization of serum-based growth factors as chemo attractants. 5 nM EGF was used as additional stimulus, when needed. Spheres were followed carefully up to four days during which the extent of the outgrowth from the spheres was carefully followed. Images were taken with Olympus 1 × 71 light microscope supplemented with Cell P software. For each of 5–6 spheres per treatment, the 10 greatest distances traveled by invading cells were estimated in ImageJ and a mean outgrowth was calculated for each treatment.

### Immunofluorescence microscopy

For lapatinib-treatment, cells were seeded on glass coverslips in 6-well culturing plates (NUNC, Thermo Scientific) 24 h prior to treatment. For mimic, LNA and siRNA transfections, cells were transfected and seeded onto coverslips simultaneously. For fixation, cells were washed twice with PBS, incubated for 20 min. with 3.7% formaldehyde at room temperature, incubated with methanol for 3 min. at −20 °C, and finally washed twice with PBS.

Immunofluorescent staining was performed as described before^14^. Primary antibodies used were LAMP2 (Abcam ab25631, 1:400) and α-tubulin (Abcam ab15246, 1:600). Images were taken with a LSM700 laser scanning confocal microscope (Carl Zeiss, Inc, Dublin, CA, USA) at 63× magnification. Quantification of the lysosomal distribution was done as described before^14^. For quantification, 5 images per treatment were analyzed in each independent experiment and classified the lysosomal distribution in 8–15 cells per image.

### Cell survival assays

Cells were plated in a 96-well plate (5000 cells/well) or a 6-well plate (300,000 cells/well) and allowed to attach for 18 h before indicated treatment (Lapatinib dose-response) or reverse transfection (Let-7 mimic), respectively. After 24 h (Lapatinib dose-response) or 72 h (Let-7 mimic transfection), the plates were stained with 0.333 µg/ml propidium iodide (Sigma-Aldrich, P4864) and 0.1 µg/ml Hoechst 33342 (Life Technologies, H1399) for 10 min at 37 °C to label dead and total cells, respectively. Samples were analyzed with the Celigo Cell Imaging Cytometer (Brooks) to image the entire well surface and quantified with the Celigo software.

### Computational analysis

Computational analysis was carried out with the Anduril framework (PMID: 20822536). RNA and microRNA sequencing data was downloaded from the Cancer Genome Atlas (TCGA) for 752 unique breast tumors for which both measurement types were available (PMID: 23000897). Both data were level 3 and had been pre-processed by TCGA as described in (PMID: 23000897). In addition, the tumor stage information was downloaded for each sample. For RNAseq data, the number of reads per kilobase per million (RPKM) was extracted and these counts were transformed to log2 scale. Before the logarithmic conversion, 1 was added to each count to avoid numerical errors. For miRNA data the number of reads per million miRNA mapped was used and similary to RNAseq they were converted to base two logarithmic scale. Correlation was calculated with the Spearman method.

### Accession numbers

The TSP study accession number in the database of Genotype and Phenotype (dbGaP) for the TCGA study used here is phs000569.v1.p7 (breast cancer).

### Statistics

Data are presented as mean ± SD of the number of experiments stated in figure legends. Data were analyzed by unpaired two-tailed Student’s t-test with Welch correction or as described in the figure legends. Statistical analysis was done using GraphPad Prism version 6.0e for Mac OS X (GraphPad Software). In all cases, *P* < 0.05 was considered statistically significant. The statistical significance is illustrated with *p*-values; *p < 0.05; **p < 0.01; ***p < 0.001; and ****p < 0.0001. Samples were only excluded from data if technical issues were evident.

## Electronic supplementary material

Supplemental Figure 1

Supplemental Figure 2

Supplemental material
