# Supplementary figures and images for: Let-7 microRNA controls invasion-promoting lysosomal changes via the oncogenic transcription factor myeloid zinc finger-1
Source: Oncogenesis. 2018 Feb 3;7(2):14. doi: 10.1038/s41389-017-0014-6 (PMC5833801; doi:10.1038/s41389-017-0014-6)

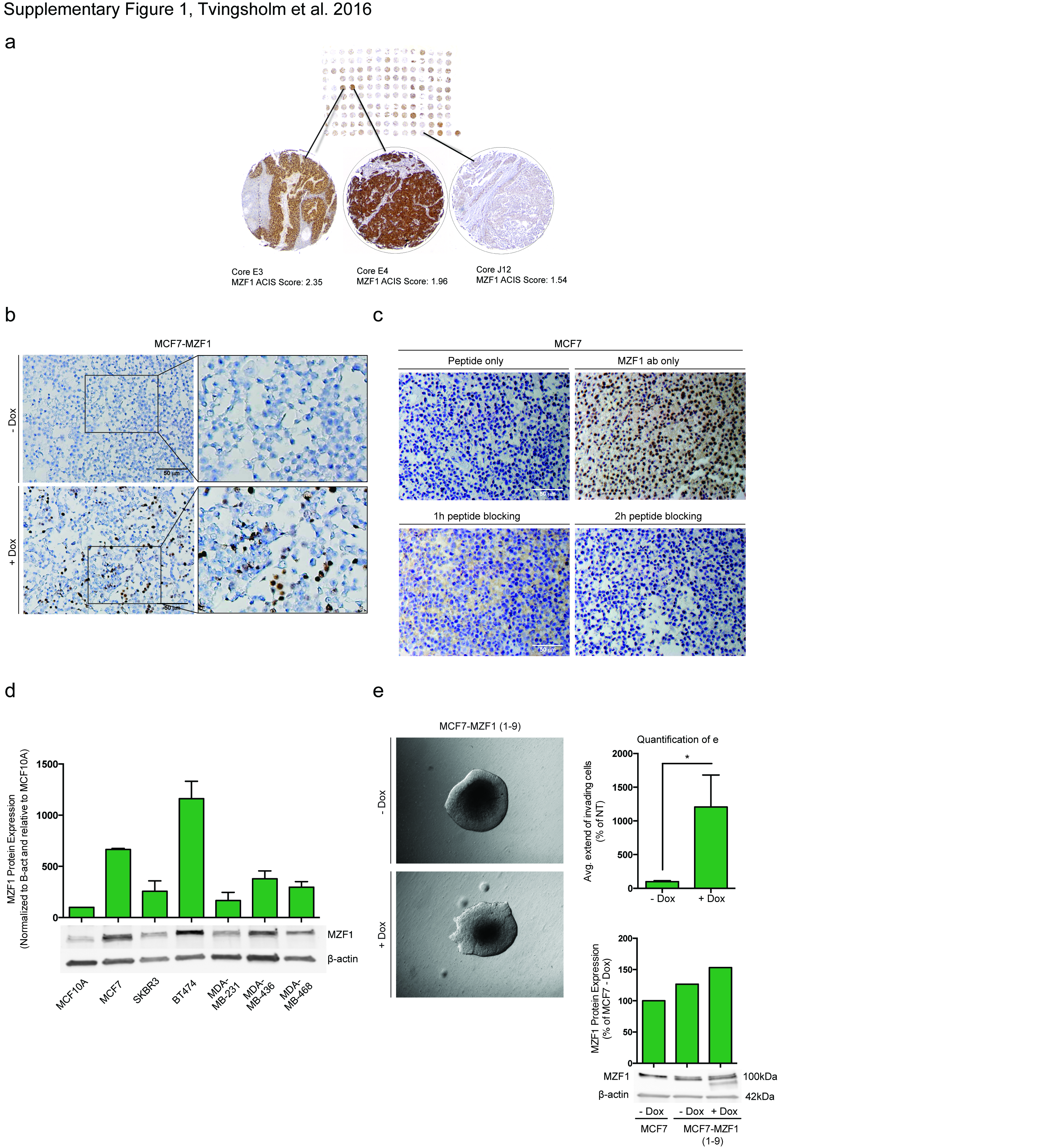

Supplement: Supplementary file 1 — Supplemental Figure 1 [file 41389_2017_14_MOESM1_ESM.tif]

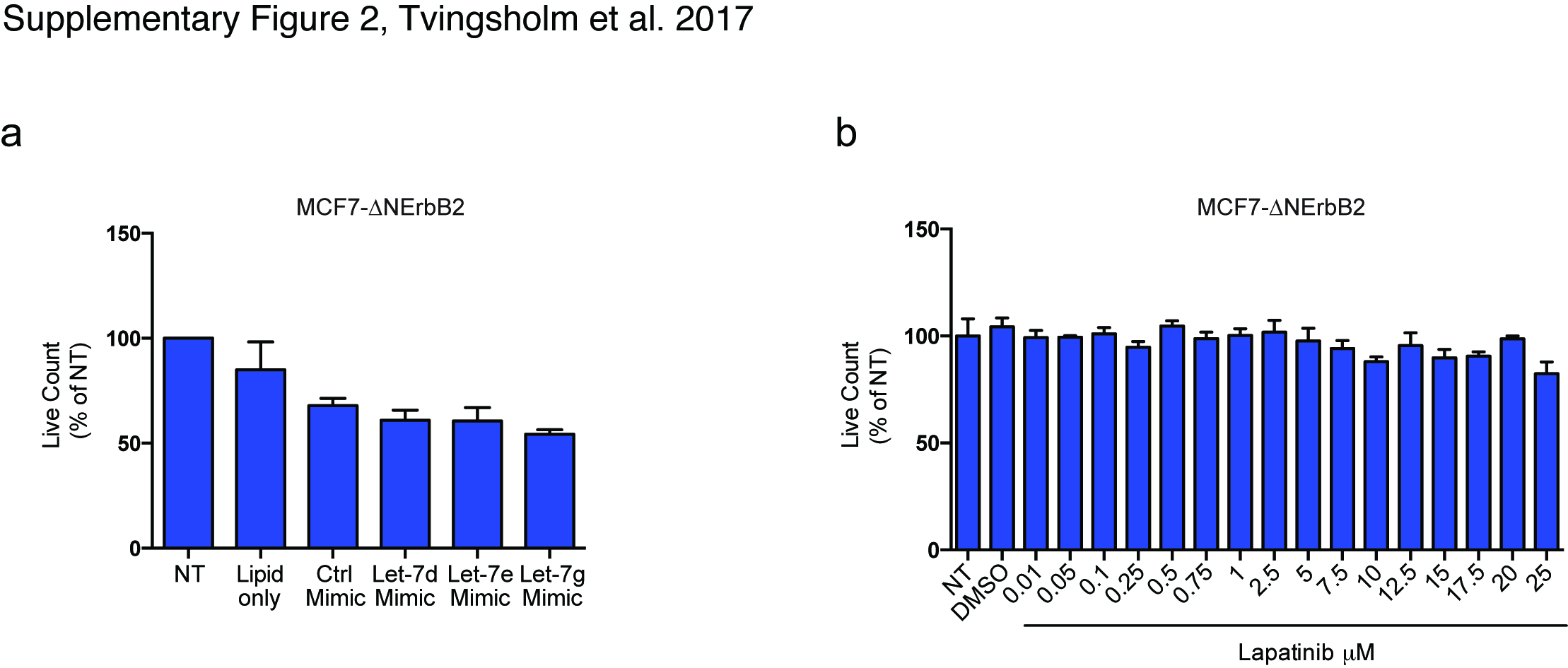

Supplement: Supplementary file 2 — Supplemental Figure 2 [file 41389_2017_14_MOESM2_ESM.tif]
