# Supplementary material for: Let-7 microRNA controls invasion-promoting lysosomal changes via the oncogenic transcription factor myeloid zinc finger-1
Source: Oncogenesis. 2018 Feb 3;7(2):14. doi: 10.1038/s41389-017-0014-6 (PMC5833801; doi:10.1038/s41389-017-0014-6)
Supplement: Supplementary file 3 — Supplemental material [file 41389_2017_14_MOESM3_ESM.docx]

Tvingsholm et al. Supplementary Figure texts

Supplementry Figure S1. Immunohistochemistry controls for the MZF1 antibody.

(**a**) Illustrative figure of the TMA setup and scoring of tissue cores using ACIS-assisted analysis. Three representative tissue cores from TMA BRC 1501 with high nuclear (core E3), high cytoplasmic (core E4) and low (core J12) MZF1 immunoreactivity are highlighted. (**b**) IHC analysis of the MZF1 protein expression in non-induced (-Doxycycline) and induced (+Doxycycline) MCF7-MZF1 cells with 0.25 µg/mL (1:4000) MZF1 ab (brown). Sections were counterstained with hematoxylin (blue) and images were taken with 40x magnification. (**c**) IHC analysis of MZF1 staining after blocking of the MZF1 ab with an immunizing peptide for 1 and 2h. FFPE MCF7 cells were incubated with 2.5 µg/mL blocking peptide only, 0.5 µg/mL (1:2000) MZF1 ab (brown) only and 0.5 µg/mL MZF1 ab and 2.5µg/mL blocking peptide solution together (1:5 ratio) for 1 and 2h. Sections were counterstained with hematoxylin (blue) and images were taken with 40x magnification. (**d**) Immunoblot analysis of MZF1 expression in MCF10A and breast cancer cell lines MCF7, SK-BR-3, BT474, MDA-MB-231, MDA-MB-436 and MDA-MB-468. β-actin was used to control for equal loading. (**e**) Invasion of single cell clone MCF7-MZF1 1-9 in 3D Matrigel in the absence (-Dox) or presence of doxycyline (+Dox). Images were taken in 10x magnification. Quantification of the average extend of outgrowth in **e** was done using ImageJ. For 4 spheres per treatment, the 10 greatest distances traveled by invading cells were estimated and a mean outgrowth was calculated for each treatment. Data presented is an average of 2 independent experiments and as % of non-induced control (-Dox) and the comparison of -Dox and +Dox in MCF7-MZF1 1-9 cells is assigned with * indicating p < 0.05 in a Welch’s t-test. Immunoblot analysis shows MZF1 induction in MCF7-MZF1 1-9 +Dox when comparing with MCF7–Dox and MCF7-MZF1 -Dox. β-actin was used as a control of equal loading.

Supplementary Figure S2.

(a) Survival of MCF7-p95ΔNErbB2 cells after 72h transfection with lipid only and 20nM of non-targeting control, let-7d, let-7e or let-7g mimic. Error bars, SD for two independent triplicate experiments.

(b) Survival of MCF7-p95ΔNErbB2 cells after 24h treatment with DMSO (Lapatinib vehicle) or indicated concentrations of lapatinib presented as the % of non-treated control cells (NT). Error bars, SD for two independent triplicate experiments.
